# Levels, Distribution, and Health Risks of Polycyclic Aromatic Hydrocarbons in Four Freshwater Edible Fish Species from the Beijing Market

**DOI:** 10.1100/2012/156378

**Published:** 2012-12-31

**Authors:** Wen-Jing Wu, Ning Qin, Wei He, Qi-Shuang He, Hui-Ling Ouyang, Fu-Liu Xu

**Affiliations:** MOE Laboratory for Earth Surface Processes, College of Urban and Environmental Sciences, Peking University, Beijing 100871, China

## Abstract

We first estimated the content of polycyclic aromatic hydrocarbons (PAHs) in the brain, liver, bladder, roe, and muscle of four species of edible freshwater fish from the Beijing market. The distribution characteristics of PAHs in these tissues and organs were analyzed to determine their health risks to humans. The results showed that the residual levels of wet weight and lipid-normalized weight **∑**PAHs in various tissues of these fish ranged from 0.51 ng*·*g^−1^ to 28.78 ng*·*g^−1^ and from 93.62 ng*·*g^−1^ to 8203.43 ng*·*g^−1^, respectively. The wet weight contents of **∑**PAHs were relatively higher in the brain and lower in the liver and muscle. But the differences were not significant. And the differences of lipid-normalized weight PAHs were significant, which in the bighead carp were found significantly the highest, followed in crucian carp, and the lowest in grass carp and carp. The contents of **∑**PAHs were the highest in the liver and the lowest in the brain. In the tissues with a higher lipid content, higher residual levels of PAHs were found. The carcinogenic risks for humans from residual **∑**PAHs in the various fish tissues were far below 10^−5^.

## 1. Introduction

Polycyclic aromatic hydrocarbons (PAHs) are a typical form of persistent organic pollutants with a wide range of distribution in various environmental media in China, particularly in the northern part of the country [1]. The emission sources of PAHs in the environment mainly include fossil fuels, wood fuels [2–4], oil spills [5], and metal smelting, among others [6, 7]. Hydrophoby and low water-solubility are two typical characteristics of PAHs [8, 9]. In addition, lipid solubility, carcinogenicity, and mutagenicity will increase when the number of rings grows larger [10]. Although PAHs will rapidly degrade once they access the body of a fish [11], research has documented that residuals of PAHs were found in the tissues and organs of various species of fish [11, 12]. 

PAHs can affect human health through various routes of exposure, such as air, food, and water, as well as in- and outdoor ambient soil and dust; and the populations' Chronic Daily Intake (CDI) of PAHs through various routes mainly depends on the PAHs levels in exposure medium, the time-behavior patterns, and physiological characteristics of human bodies [1, 13–16]. It was reported that, in the United States, the PAHs exposure through food consumptions accounted for 96% for the aged 19–50 nonsmokers [15]. In Montreal, Canada, the children's exposure to PAHs through food consumptions accounted for 93% to 97% [16]. However, in Tianjin, China, populations' exposure to PAHs through dietary intake, respiratory, and skin contact accounted approximately for 75%, 20%, and 5% of the total exposure, respectively [1]. These reports suggest that dietary intake is a predominant route of PAHs exposure to harm human health. Because fish plays a key role in the food chain in comparison to other types of food media of PAHs intake such as vegetables [17], it is a primary intermediary through which these pollutants access the human body, even though fish only constitutes approximately ten percent of the dietary intake of humans [18]. Therefore, the residual level of PAHs in fish, and particularly in edible fish, has a great effect on human health. This paper will estimate the content of PAHs in various tissues and organs of four commonly found edible fish species at the Beijing market. These fish tissues and organs, although not all harvested by all peoples across the world, are all ingested by the Chinese people to a larger or smaller extent, according to the dietary habit. In addition, we will show how the content is distributed in the tissues and organs of the fish and how it is distributed across the various species that we are studying. We will also discuss the potential risks for human health through an analysis of wet weight contents and lipid-normalized contents.

## 2. Materials and Methods

### 2.1. Sample Collection

50 specimens from each of four commonly consumed freshwater fish species, namely, crucian carp (*Carassius auratus*), bighead carp (*Aristichthys nobilis*), carp (*Cyprinus carpio* L), and grass carp (*Ctenopharyngodon idellus*) were collected from the Yuegezhuang wholesale market, which is the largest aquatic product market in the Beijing region. Five tissues and organs from these specimens, including the brain, liver, bladder, roe, and a muscle mixture from the dorsal and chest were sampled. To eliminate individual diversity, tissues from five specimens of each species were combined into one sample. All of the samples were freeze-dried for three to four days after weighing and then preserved in the desiccator prior to analysis. The general physiological information of the fish is shown in Table 1.

### 2.2. Sample Pretreatment

The freeze-dried fish tissue samples, each weighing approximately 3 grams, were first ground with anhydrous sodium sulfate. The samples were then Soxhlet extracted with 100 mL of mixed solvent of dichloromethane and n-hexane (v : v, 4 : 1) for 24 h at 60°C. The extracted mixed solvent was then transformed into an n-hexane solvent and concentrated into 3 mL. The next step was the liquid-liquid extraction followed by Haruhiko's procedure [19]. The lipid content within the tissues and organs was measured using the quality-subtraction method. A silica gel column was used for the sample cleanup. The cleanup column was eluted with 50 mL of n-hexane followed by 50 mL of a 3 : 2 mixture of n-hexane and dichloromethane. The eluate collected from the silica column during cleanup was concentrated into 0.2 mL using a vacuum rotary evaporator. The samples were sealed in vials and stored at −4°C prior to analysis.

### 2.3. Sample Analysis

16 priority PAHs identified by the USEPA including naphthalene (Nap), acenaphthylene (Acy), acenaphthene (Ace), fluorene (Flo), phenanthrene (Phe), anthracene (Ant), fluoranthene (Fla), pyrene (Pyr), chrysene (Chr), benzo[a]anthracene (BaA), benzo[b]fluoranthene (BbF), benzo[k]fluoranthene (BkF), benzo[a]pyrene (BaP), indeno[1,2,3-cd]pyrene (IcdP), benzo[ghi]perylene (BghiP), and dibenz[a,h]anthracene (DahA) were analyzed in this study. The analysis was conducted using an Agilent 6890 GC, coupled with an Agilent 5973 mass spectrometer and a 7683 autosampler (Agilent Technology). An HP-5 MS capillary column with 30 m × 0.25 mm × 0.25 *μ*m film thickness was used. High-purity helium was used as the carrier gas. Samples of 1 *μ*L were injected using the splitless mode at a flow rate of 1.0 mL/min. The temperatures of the injection port and ion source were maintained at 220°C and 280°C, respectively. GC temperature was programmed from an initial 60°C at 6°C/min up to 260°C, with a final holding time of 20 min. The mass spectrometer was operated in scan mode with an electron impact ionization of 70 eV. The quality range is from 45 to 600 amu, an electron multiplier voltage of 1288 V and an ion source at 280°C. 

### 2.4. Quality Control

Prior to the sample analysis, a mixed stock standard with 16 PAHs (PAH-Mixture, 610/525/550, Chem. Service Co.) was used to make the standard curve with the concentration of 1 ppb, 10 ppb, 100 ppb, and 1000 ppb. The procedural blank was determined by going through the extraction and cleanup procedures using glass beads instead of fish samples. Recoveries of PAHs were determined by spiking fish samples with standards at both higher and lower concentrations. Recovery rates and detection limits (dry weight data and PAH content in freeze-dried samples of unit mass) for PAHs in fish samples are shown in Table 2. With regard to sample data that are lower than detection limits, one-third of this value was counted in the statistics (BkF is an exception because of its high detection limits, and therefore detected value was used). Dry weight content was transformed into wet weight content and lipid-normalized content (dry weight content divided by the percentage of lipid in the dry weight sample). 

### 2.5. Data Processing

The software used was SPSS 13.0. The Shapiro-Wilk test was used to estimate data normality, under which we found that PAH data from the wet weight basis fit neither normal distribution nor logarithmic normal distribution. Moreover, PAH data from the lipid-normalized weight basis did not fit into the normal distribution but it did, to an extent, present as a logarithmic normal distribution. A log-transformation was performed to ensure the normality of the distribution of data in all tissues. A two-way ANOVA (analysis of variance) was conducted to detect differences in data among tissues and species. The relationship between the data was determined by Pearson's sample correlation, and when the value of *P* was below 0.05, the linear regression was regarded as significant.

## 3. Results

### 3.1. Wet Weight Contents and Composition Patterns of 16 Types of PAHs

**Figure 1:**
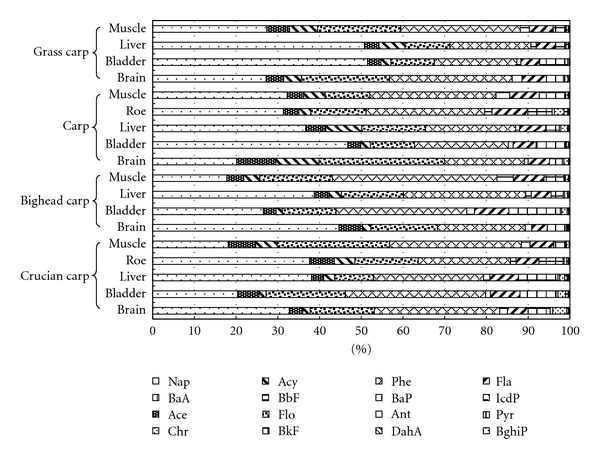
Percentage composition of 16 priority PAHs congeners in the fish from Beijing market.

**Figure 2:**
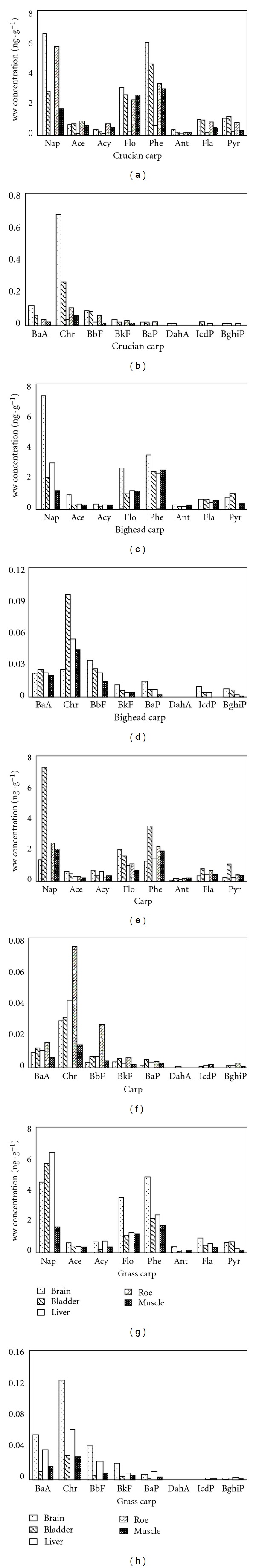
Distribution pattern of PAH congeners in the tissues and organs of four fish species.

Residual levels of sixteen types of PAHs (*∑*PAHs) on a wet weight basis in various tissues of the four types of fish species ranged from 0.51 ng·g^−1^ to 28.78 ng·g^−1^, with a mean of 10.17 ± 0.67 ng·g^−1^. Figures 1 and 2 show percentages and contents of all sixteen types of PAHs in various tissues of the four fish species, respectively. Low molecular weight PAHs (LMWPAHs) with two or three rings (Nap, Acy, Ace, Flo, Phe, Ant, Fla) took the largest share as 85.4%*∼*96.6%. The proportion of middle molecular weight PAHs (MMWPAHs) with four rings (Pyr, BaA, Chr, BbF, BkF) ranged from 3.3% to 14.4%; Pyr dominated this segment with a share of 2.2% to 12.3%. High molecular weight PAHs (HMWPAHs) with five rings (BaP, IcdP, DahA, BghiP) only accounted for a share of approximately 0 to 0.5 percent. Residual levels of Nap, Flo, and Phe were high in the tissues of the various species, of which Nap measured approximately 32%, Phe approximately 27%, and Flo approximately 17%. Correlational studies also found that the main forms of PAH residuals in living creatures in general were PAHs with two or three rings [18, 20–22]. By contrast, general residual levels of PAHs with four rings were lower and those of PAHs with five rings were extremely low. For example, the proportion of low molecular weight PAHs, middle molecular weight PAHs, and high molecular weight PAHs residuals in fish from Italy (Adriatic Sea) were 62%, 37%, and 1%, respectively [23]. A similar tendency in the levels of PAHs residuals was also found by Binelli [18] in research on European shellfish. Vives found that Phe contents in salmon liver from lakes in Europe and Greenland were as high as 52% [11]. He also found high levels of Phe residuals in various environmental media, such as water, suspended matter, and deposit sediment. 

The content of Nap, Flo, and Phe in various tissues of all four species in our study is relatively high, as shown in Figure 2, with the exception of the crucian carp liver. The distribution of Nap varied significantly among different tissues, ranging from 1.1 ng·g^−1^ *∼*7.2 ng·g^−1^ and was generally lower in the muscle. Flo levels were higher in the brain than in other tissues (2.0 ng·g^−1^ *∼*3.5 ng·g^−1^). With regard to the levels of Phe content, higher values were in the brain of crucian carp, bighead carp, and grass carp and in the bladder of carp (3.5 ng·g^−1^ *∼*6.0 ng·g^−1^). The contents of PAHs in the liver of crucian carp were significantly lower than in other tissues. Residual levels of Chr were the highest among all the four ring PAHs (0.03 ng·g^−1^ *∼*0.66 ng·g^−1^). However, residual levels of HMWPAHs were extremely low.

In comparison with similar studies, the wet weight PAH16 contents in the freshwater fish of this study are lower than those found in most other studies, with a mean level of 10.17 ng·g^−1^ ranging from 0.51 ng·g^−1^ to 28.78 ng·g^−1^. For instance, the levels of PAH on a wet weight basis were found to range from 19.7 ng·g^−1^ to 154.3 ng·g^−1^ in Bolti fish and mallet fish collected from markets in Ismailia city, Egypt [12]. The contents of *∑*PAHs in fish from Lake Victory in Africa were between of 0.035 ng·g^−1^ and 3.934 ng·g^−1^ [24]. And *∑*PAHs contents in Mullus barbatus from the Sicily Channel in Italy had a mean of 26.47 ng·g^−1^  ±  34.16 ng·g^−1^ [25]. In China, PAHs content levels in the freshwater and marine fish collected from the Hong Kong market varied from 15.5 ng·g^−1^ to 118 ng·g^−1^ (ww) [26], while PAHs content levels in freshwater fish from the Pearl River delta were 30.94–410.06 ng·g^−1^ (ww) [27].

### 3.2. PAHs Distribution in Various Fish Tissues on a Wet Weight Basis

The mobility of PAHs in the environment is normally determined by molecular weight. Related researches have shown that the mobility of low molecular weight PAHs in the atmosphere is relatively higher than that of middle and high molecular weight PAHs [28, 29]. However, middle and high molecular weight PAHs usually have higher carcinogenicity and mutagenicity [28, 29]. Considering this, we categorized PAHs by the number of their rings. PAHs with two or three rings are defined as low molecular weight PAHs (LMWPAHs). Those PAHs with four rings belong to middle molecular weight PAHs (MMWPAHs), and those with five rings belong to high molecular weight PAHs (HMWPAHs). Residual levels of PAHs on a wet weight basis in various fish tissues are illustrated in Table 3. 

**Figure 3:**
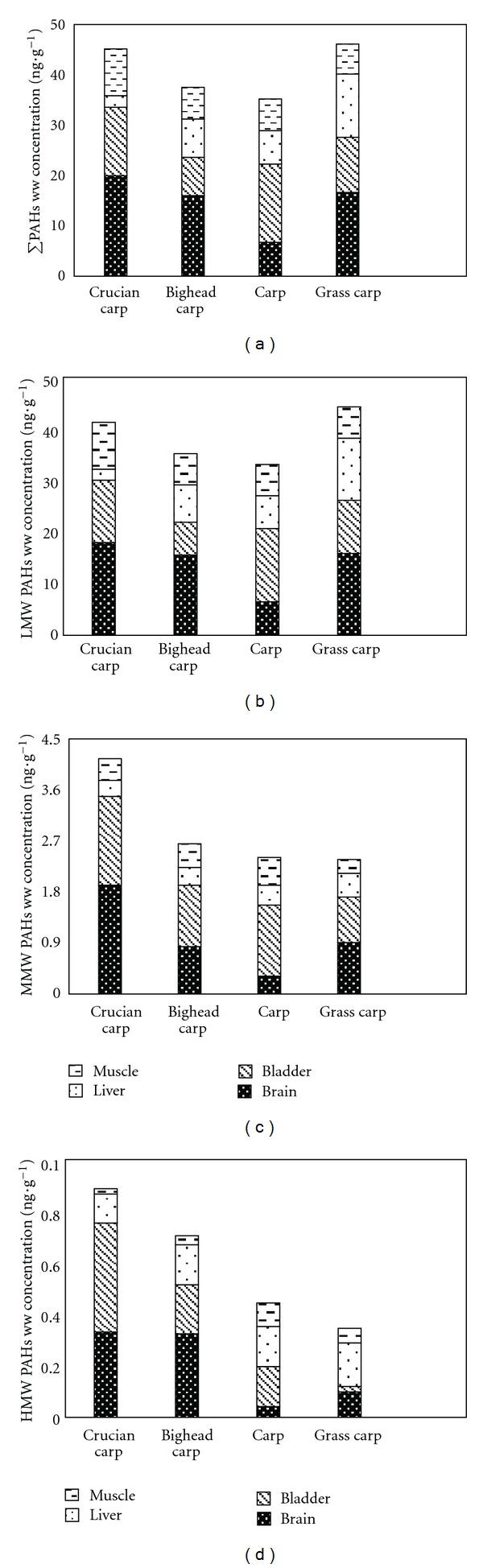
Wet weight contents of (a) total PAHs (PAH16), (b) LMW-PAHs, (c) MMW-PAHs, and (d) HMW-PAHs in four fish species.

The distribution of LMWPAHs and total PAHs share a similar pattern, as shown in Figure 3. Residual levels of PAHs on a wet weight basis were found slightly higher in crucian carp and grass carp and relatively lower in bighead carp and carp. Nevertheless, the variances of PAHs residuals on a wet weight basis among the species studied were not wide. The distribution of MMWPAHs on a wet weight basis was found higher in crucian carp and was approximately equal in the other three species. A decreasing pattern of HMWPAHs distribution was found among crucian carp, bighead carp, carp, and grass carp, respectively. However, these differences were not significant at a 95% confidence level (*P* > 0.05), under the two-way ANOVA.

**Figure 4:**
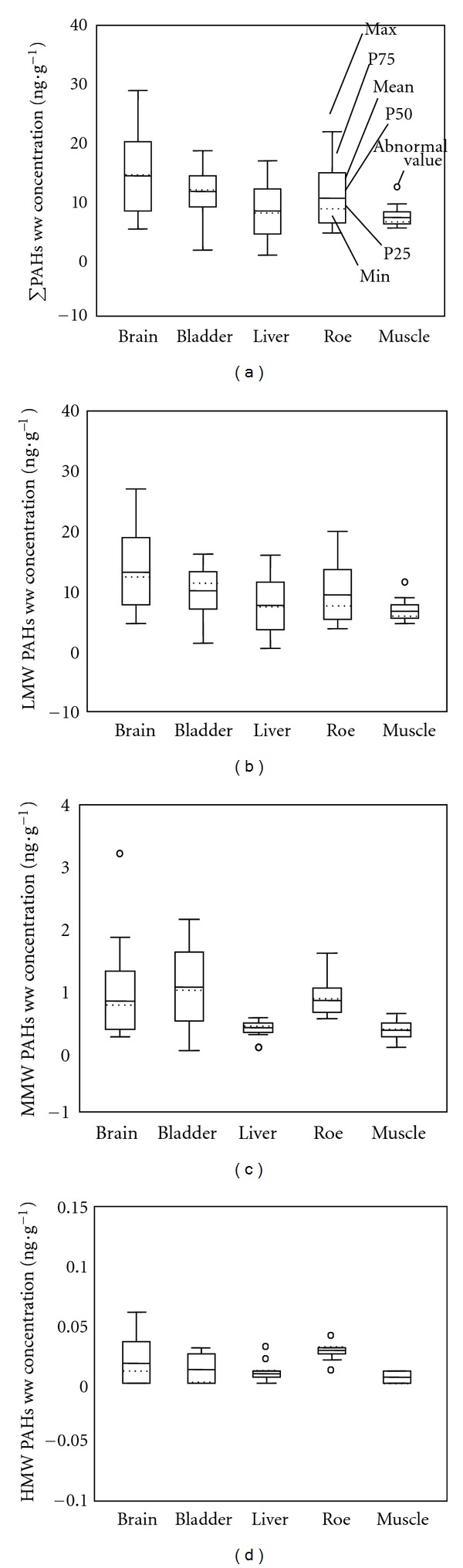
Wet weight contents of (a) total PAHs (PAH16), (b) LMW-PAHs, (c) MMW-PAHs, and (d) HMW-PAHs in four fish tissues.

Residual PAH levels in various tissues are shown in Figure 4. The distribution of total PAHs and LMWPAHs was found to be highest in the brain, lower in the bladder and roe, and the lowest was found in the liver and muscle. With regard to the distribution of MMWPAHs, the highest levels of content were found in the bladder, lower levels in the brain and roe, and the lowest were found in the liver and muscle. Finally, the distribution of HMWPAHs was found to be highest in the roe, lower in the brain, and the lowest was found in the bladder, liver, and muscle. The result of the two-way ANOVA is shown in Table 4, which showed that residual levels of PAHs on a wet weight basis were tested to be significant under a 95% confidence level (*P* < 0.05). 

### 3.3. PAH Distribution in Various Fish Tissues on a Lipid-Normalized Weight Basis

**Figure 5:**
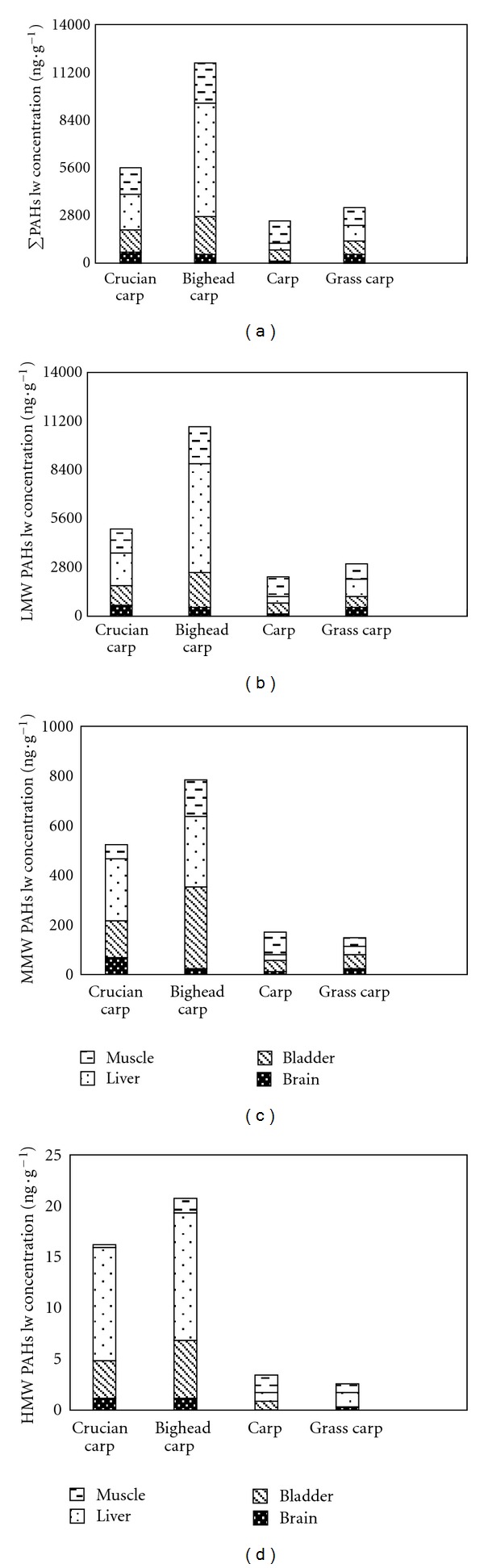
Lipid normalized contents of (a) total PAHs (PAH16), (b) LMW-PAHs, (c) MMW-PAHs, and (d) HMW-PAHs in four fish species.

The distribution of sixteen types of PAHs in various fish tissues on a lipid-normalized weight basis shown in Table 5 ranges from 93.62 ng·g^−1^ to 8203.43 ng·g^−1^, with a mean value of 1204.18 ng·g^−1^ ± 144.16 ng·g^−1^.  Figure 5 shows the differences on a lipid-normalized weight basis of the various species. Residual levels of PAHs on a lipid-normalized weight basis were found to be highest in bighead carp, lower in crucian carp, and the lowest in carp and grass carp. The distribution of total PAHs, LMWPAHs, and MMWPAHs shared similar patterns. According to the two-way ANOVA results (Table 6), the residual level of PAHs on a wet weight basis was tested to be significant under a 95% confidence level (*P* < 0.05). This is most likely related to the different feeding habits of the various species. Bighead carp is a filter feeder, feeding on zooplankter, leading to the highest residual levels of PAHs on a lipid-normalized weight basis. Crucian carp and carp are omnivorous fish and therefore the residual level of PAHs on a lipid-normalized weight basis was found to be lower. Grass carps are herbivorous, and they were found to contain the lowest residual level of PAHs on a lipid-normalized weight basis.

**Figure 6:**
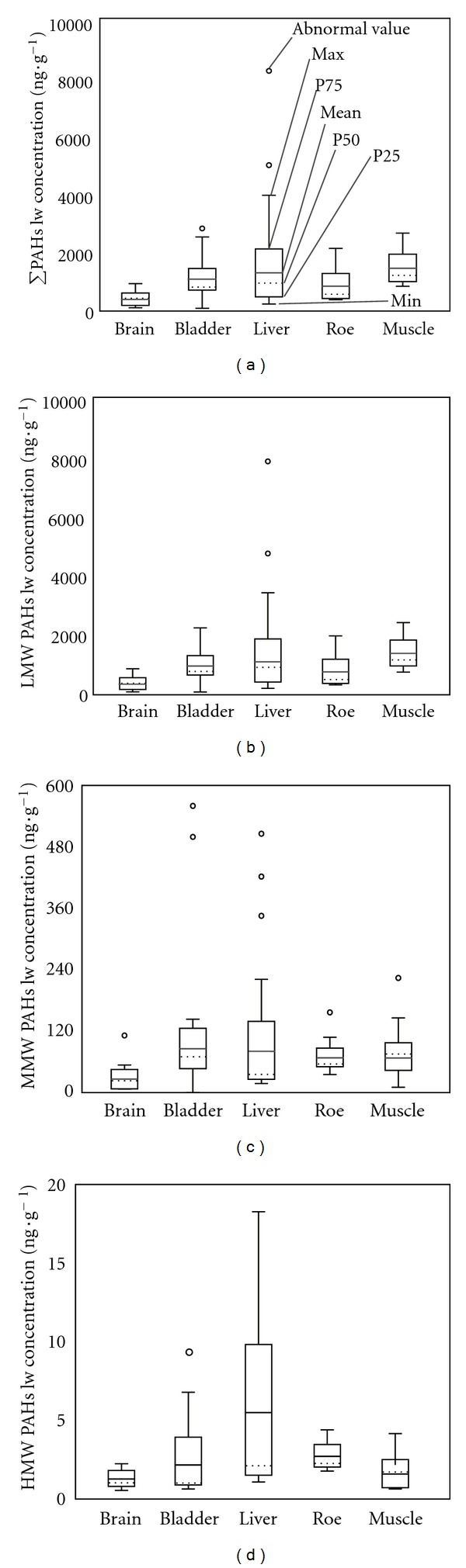
Lipid-normalized contents of (a) total PAHs (PAH16), (b) LMW-PAHs, (c) MMW-PAHs, and (d) HMW-PAHs in four fish tissues.

The different residual levels of PAHs on a lipid-normalized weight basis in various tissues and organs are shown in Figure 6. In general, the distribution of total PAHs, LMWPAHs, MMWPAHs, and HMWPAHs shared a similar tendency, which is that residual levels of PAHs were the highest in the liver, lower in the muscle, bladder, and roe, and lowest in the brain. A one-way analysis of variance reflected that the differences of PAH residual levels on a lipid-normalized weight basis in various tissues were significant at a 95% confidence level (*P* < 0.05). An ideal illustration for this is the liver block phenomenon, which holds that pollutants in the living body will integrate with related proteins to form a compound. This compound, consisting mainly of various cytochromes of P450, will subsequently be transferred into the liver, causing pollutants to accumulate and concentrate there [30]. In comparison, the low concentration of pollutants in the brain is thought to be related to the blood-brain barrier, which consists of a layer of endothelial cells that exists in many organisms [31]. The main biological function of this blood-brain barrier is to resist various pathogens and poisonous substances. The selective entry of molecules in the brain lies in its structural characteristics of being both highly complex and highly ordered. This can ensure an accurate identification of outgoing substances in its biological, chemical, and physical properties, as well as in its spatial structure [32].

### 3.4. Relation between Lipid Content and Residual Level of PAHs in Fish

Table 7 illustrates the related coefficient and significance levels between lipid content and the residual level of PAHs in the specimens we studied. The relation between lipid content and residual level of total PAHs, LMWPAHs, and MMWPAHs was found significant at a 0.01 confidence level. However, the correlation between lipid content and the residual level of HMWPAHs is lower (*R* = 0.249, *P* = 0.051). In tissues with high lipid content, higher residual levels of PAHs were also found. For instance, lipid content is as high as 22.5%*∼*35.0% in the brain and 4.6%*∼*6.8% in the roe, and residual level of PAHs in these tissues was also high (see Figure 3).

### 3.5. Risks to Human Health from PAHs in Fish

Our research adopted a screen value (SV) to assess the health risks of PAHs to humans from eating these four fish species. Screen value is defined as the concentration of chemicals in edible tissue that are a potential public health concern. The SV indicator is calculated according to the following formula [26, 33–35]:

(1) $$\begin{array}{c} \text{SV}=\frac{\left[\left(\text{RL}/\text{SF}\right)*\text{BW}\right]}{\text{CR}}, \end{array}$$

where SV is the screening value (*μ*g·g^−1^) that is used as a threshold value against the residue level in similar tissue collected from the environment; RL is the maximum acceptable risk level (dimensionless, using 10^−5^); SF is the oral slope factor (*μ*g·g^−1^·d^−1^)and the SF value of BaP is 7.3 (*μ*g·g^−1^·d^−1^)^−1^; BW is body weight (kg) and an average of 70 kg is used for the calculations; and CR refers to the consumption rate (g·d^−1^) and is substituted by the USEPA standard value for the average intake rate of fish, which is 142.2 g·d^−1^. 

 When the carcinogenic risk is 10^−5^, the calculated SV threshold is 0.67 ng·g^−1^ (wet weight, the potency equivalent concentration) [36–39], which is far higher than the wet weight contents of PAHs in various tissues and organs acquired from this research. Therefore, we can clarify that the carcinogenic risk from the four main fish species found in the Beijing market is significantly lower than 10^−5^ (Table 8).

In addition, BaP equivalent concentrations of PAHs in the fish measured in this research were also below the national standards limiting pollutants, in which BaP equivalent concentrations are suggested below 5 ng·g^−1^ for grain and meat and 10 ng·g^−1^ for vegetables [35, 40].

## 4. Conclusions

The residual levels of PAHs in fish on a wet weight basis ranged from 0.51 ng·g^−1^ to 28.78 ng·g^−1^, while the residual levels of PAHs on a lipid-normalized basis ranged between 93.62 ng·g^−1^ and 8203.43 ng·g^−1^. LMWPAHs took a dominant percentage of the total of the PAH residuals.

The residual levels of PAHs in fish on a wet weight basis had no significant differences among various fish species but did show differences among the various fish tissues. The highest residual levels of total PAHs and LMWPAHs were found in the brain, they were lower in the roe and bladder, and they were the lowest in the liver and muscle. With regard to residual levels of MMWPAHs, the highest value was found in the bladder, lower values in the brain and roe, and the lowest values in the liver and muscle. For HMWPAHs, the highest value was found in the roe, lower values in the brain, and the lowest values in the bladder, liver, and muscle. 

The residual levels of PAHs on a lipid-normalized weight basis had significant differences both for various fish species and for various fish tissues. In all of the four fish species, the highest total residual levels of PAHs was found in bighead carp, lower levels in crucian carp and the lowest values in carp, and grass carp. As for the five tissues, the highest residual levels of PAHs were found in the liver, lower levels in the muscle, roe, and bladder, and the lowest levels in the brain. No significant differences were found for PAH distribution in HMWPAH, MMWPAHs, and LMWPAHs.

The potency equivalent concentration of PAHs on a wet weight basis in various tissues was found to be lower than the SV value from USEPA (0.67 ng·g^−1^, wet weight). Therefore, its carcinogenic risk for humans was far lower than 10^−5^.

## Figures and Tables

**Table 1:** Fish species and their characteristics collected from a local market in Beijing.

Common name	Scientific name	Feeding mode	Average length (cm)	Average weight (g)	Lipid contents
Crucian carp	*Carassius auratus *	Omnivorous	20	250	0.6%~22.5%
Bighead carp	*Aristichthys nobilis *	Filter feeder	34	750	0.6%~24.1%
Carp	*Cyprinus carpio *	Omnivorous	25	450	2.4%~35.0%
Grass carp	*Ctenopharyngodon idellus *	Herbivorous	34	650	2.9%~26.8%

**Table 2:** Recovery rates and detection limits for PAHs in fish samples.

	Nap	Ace	Acy	Flo	Phe	Ant	Fla	Pyr	BaA	Chr	BbF	BkF	BaP	DahA	IcdP	BghiP
Recovery rates	63.7%	90.0%	97.8%	116.0%	114.0%	124.3%	139.8%	145.5%	140.5%	108.6%	138.7%	133.4%	149.6%	132.6%	130.9%	119.6%
Detection limits (ng·g^−1^)	1.02	0.61	0.65	0.65	0.60	0.41	0.38	0.38	0.51	0.70	1.14	0.39	0.45	0.30	0.48	0.34

**Table 3:** Residual levels of PAHs (wet weight) (ng·g^−1^) in freshwater fish samples.

PAHs	Species	Brain	Bladder	Liver	Roe	Muscle
*∑*PAHs	Crucian carp	19.77 ± 4.71	13.63	2.28 ± 1.65	14.90 ± 9.33	9.40 ± 1.94
	Bighead carp	16.10 ± 9.59	7.52 ± 1.65	7.63 ± 2.63	—^b^	6.26 ± 0.62
	Carp	6.65 ± 1.71	15.46 ± 2.33	6.74 ± 1.82	7.84 ± 2.28	6.34 ± 1.63
	Grass carp	16.50 ± 4.80	11.01 ± 5.22	12.57 ± 2.47	—	6.13 ± 0.62

LMWPAHs	Crucian carp	17.81 ± 5.74	12.00	2.01 ± 1.41	13.79 ± 8.81	9.04 ± 1.78
	Bighead carp	15.24 ± 9.01	6.42 ± 0.97	7.29 ± 2.53	—	5.87 ± 0.47
	Carp	6.32 ± 1.70	14.21 ± 2.16	6.35 ± 1.78	7.06 ± 2.12	5.87 ± 1.53
	Grass carp	15.58 ± 4.34	10.22 ± 4.78	12.14 ± 2.44	—	5.89 ± 0.73

MMWPAHs	Crucian carp	1.93 ± 1.12	1.59	0.27 ± 0.25	1.06 ± 0.53	0.37 ± 0.16
	Bighead carp	0.84 ± 0.59	1.08 ± 0.77	0.33 ± 0.11	—	0.38 ± 0.17
	Carp	0.33 ± 0.017	1.23 ± 0.78	0.38 ± 0.081	0.76 ± 0.19	0.46 ± 0.093
	Grass carp	0.91 ± 0.54	0.80 ± 0.51	0.41 ± 0.096	—	0.25 ± 0.17

HMWPAHs	Crucian carp	0.033 ± 0.031	0.03	0.005 ± 0.01	0.027 ± 0.015	0.0025 ± 0.005
	Bighead carp	0.033 ± 0.017	0.018 ± 0.015	0.01 ± 0.00	—	0.0025 ± 0.005
	Carp	0.0025 ± 0.005	0.015 ± 0.013	0.01 ± 0.0082	0.028 ± 0.005	0.01 ± 0.00
	Grass carp	0.008 ± 0.0084	0.00 ± 0.00	0.013 ± 0.0082	—	0.0033 ± 0.0052

^a^Levels of PAHs are presented as mean ± standard deviation.

^b^“—” means no samples were collected.

**Table 4:** ANOVA results of wet weight-based PAH contents.

PAHs		Square	*F* value	Sig.
*∑*PAHs	Species	44.93	1.81	0.154
	Tissues and organs	161.31	6.51	0.000

LMWPAHs	Species	42.26	1.90	0.138
	Tissues and organs	132.40	5.96	0.000

MMWPAHs	Species	0.44	1.84	0.150
	Tissues and organs	1.85	7.80	0.000

HMWPAHs	Species	0.00033	2.21	0.095
	Tissues and organs	0.00072	4.78	0.002

**Table 5:** Residual levels of PAHs (lipid normalized) (ng·g^−1^ lw) in freshwater fish samples.

PAHs	Species	Brain	Bladder	Liver	Roe	Muscle
*∑*PAHs	Crucian carp	685.92 ± 163.49	1233.83	2168.78 ± 1566.04	1469.73 ± 920.61	1496.40 ± 308.88
	Bighead carp	523.01 ± 311.46	2281.04 ± 500.70	6593.50 ± 2276.79	—	2317.51 ± 228.61
	Carp	148.35 ± 38.20	665.95 ± 100.33	394.66 ± 106.71	527.30 ± 153.16	1230.44 ± 315.89
	Grass carp	81.78 ± 140.27	762.68 ± 361.76	967.79 ± 190.42	—	101.36 ± 100.69

LMWPAHs	Crucian carp	617.55 ± 199.01	1086.74	1901.88 ± 1329.31	1361.42 ± 869.41	1438.56 ± 283.38
	Bighead carp	494.86 ± 292.80	1947.79 ± 293.57	6297.75 ± 2188.34	—	2174.14 ± 172.63
	Carp	141.12 ± 38.06	612.17 ± 93.25	371.90 ± 104.54	474.34 ± 142.55	1140.44 ± 297.25
	Grass carp	454.95 ± 126.86	707.60 ± 331.12	934.95 ± 187.91	—	961.22 ± 118.39

MMWPAHs	Crucian carp	67.21 ± 38.66	143.36	255.98 ± 242.39	105.40 ± 52.52	57.43 ± 26.44
	Bighead carp	27.08 ± 19.06	327.50 ± 234.74	283.10 ± 87.72	—	141.96 ± 59.88
	Carp	7.13 ± 0.39	53.10 ± 33.71	21.87 ± 4.45	51.38 ± 12.68	88.17 ± 18.53
	Grass carp	26.54 ± 15.72	54.95 ± 35.39	31.52 ± 7.52	—	39.27 ± 27.54

HMWPAHs	Crucian carp	1.17 ± 0.73	3.73	10.92 ± 5.54	2.91 ± 1.50	0.42 ± 0.60
	Bighead carp	1.07 ± 0.49	5.74 ± 2.53	12.65 ± 0.74	—	1.41 ± 1.53
	Carp	0.10 ± 0.049	0.68 ± 0.49	0.89 ± 0.43	1.59 ± 0.26	1.82 ± 0.36
	Grass carp	0.29 ± 0.29	0.12 ± 0.095	1.32 ± 0.64	—	0.87 ± 1.35

**Table 6:** ANOVA results of lipid normalized-based *∑*PAHs contents.

PAHs		Mean square	*F* value	Sig.
*∑*PAHs	Species	11741830.31	13.93	0.000
	Tissues and organs	4989904.44	5.92	0.000

LMWPAHs	Species	9814905.47	13.18	0.000
	Tissues and organs	4410348.16	5.92	0.000

MMWPAHs	Species	88756.14	9.69	0.000
	Tissues and organs	31423.44	3.43	0.013

HMWPAHs	Species	70.79	10.50	0.000
	Tissues and organs	53.68	7.96	0.000

**Table 7:** Correlation between LMPAHs contents in dry, wet, and Ln-transformed lipid contents.

	Ln(*∑*PAHs)	Ln(LMWPAHs)	Ln(MMWPAHs)	Ln(HMWPAHs)
Pearson correlation coefficient (*R*)	0.659	0.663	0.528	0.249
Significance level (*P*)	<0.001	<0.001	<0.001	0.051

**Table 8:** Risk assessment on BaP and potency equivalent concentration of PAHs for human consumption of fish.

	Species	Brain	Bladder	Liver	Roe	Muscle
BaP	Crucian carp	5.5 × 10^−7^	4.0 × 10^−7^	1.5 × 10^−7^	4.3 × 10^−7^	1.9 × 10^−7^
	Bighead carp	4.3 × 10^−7^	2.2 × 10^−7^	2.2 × 10^−7^	—	2.1 × 10^−7^
	Carp	1.6 × 10^−7^	3.3 × 10^−7^	2.4 × 10^−7^	3.1 × 10^−7^	2.2 × 10^−7^
	Grass carp	2.7 × 10^−7^	2.7 × 10^−7^	3.3 × 10^−7^	—	3.0 × 10^−7^

PEC of PAHs	Crucian carp	1.14 × 10^−6^	7.7 × 10^−7^	1.7 × 10^−7^	6.4 × 10^−7^	2.6 × 10^−7^
	Bighead carp	6.2 × 10^−7^	3.5 × 10^−7^	3.3 × 10^−7^	—	2.4 × 10^−7^
	Carp	2.2 × 10^−7^	5.3 × 10^−7^	3.4 × 10^−7^	5.2 × 10^−7^	2.9 × 10^−7^
	Grass carp	6.1 × 10^−7^	2.3 × 10^−7^	5.0 × 10^−7^	—	2.2 × 10^−7^
